# Eldicus scores for admission to our ICU: a retrospective study

**DOI:** 10.1186/cc12415

**Published:** 2013-03-19

**Authors:** G Zani, B Gemelli, S Vitali, C Villani, R Ragazzi, S Spadaro, CA Volta, R Alvisi

**Affiliations:** 1 University of Ferrara, Italy

## Introduction

The indication for ICU admission is poorly defined and identifying patients who may benefit from it is extremely difficult. As reported in literature, the Eldicus scores require further validation in centers in different countries and with different patient populations [1]. Previous studies have demonstrated that patients with intermediate severities of illness are those that benefit from ICU treatment; instead, patients who are too well or too sick should not be admitted. There are many unmeasurable factors that influence the triage decision and can influence the subsequent outcome. The Eldicus scores could be the first triage decision rule. The aim of this study was to confirm the ability of the Eldicus scores to discriminate the survival status in our general ICU.

## Methods

We retrospectively enrolled all patients >18 years admitted to the Ferrara ICU from January to June 2012. Patients' data were recorded using Prosafe international data collection software and consulting the individual medical records. Data were chosen based on variables used in the Eldicus scores. As indicated in the Eldicus algorithm, for each patient the initial refusal score was calculated, to evaluate the too severe patients. Then we counted the final triage score, to discover whether some patients were too healthy to be admitted to the ICU. Correlation between the severity of the scores and patients' outcome was estimated using the Wilcoxon's rank-sum test.

## Results

Ninety-eight patients were finally enrolled. Data are presented as median ± SD. The SAPS II score was 33 ± 13 and was similar to those reported in the literature [1]. The Karnofsky performance status scale was 69 ± 20. The Eldicus initial score was 56 ± 30. The final score was 70 ± 48. Seventy-eight percent of the analyzed patients were alive at hospital discharge. A significant correlation between the severity scores and in-hospital outcome was found (*P *0.001). Limitations of our study were the low sample size and the fact that patients not admitted to the ICU were not considered.

## Conclusion

The Eldicus scores appear to be useful in identifying patients that may benefit more from ICU admission. Further studies should confirm the ability of these scores in helping identify those patients who really benefit from ICU admission.
